# A Systematic Review and Meta-Analysis Comparing Surgical and Nonsurgical Treatments for Excessive Gingival Display

**DOI:** 10.3390/dj12060154

**Published:** 2024-05-22

**Authors:** Mahdis Maleki, Bo Huang, Vanessa C. Mendes, Marco F. Caminiti, Yoav Finer

**Affiliations:** 1 Faculty of Dentistry, University of Toronto, 124 Edward St., Toronto, ON M5G 1G6, Canada; m.maleki@mail.utoronto.ca (M.M.); boya.huang@dentistry.utoronto.ca (B.H.); vanessa.mendes@dentistry.utoronto.ca (V.C.M.); marco.caminiti@utoronto.ca (M.F.C.); 2 Institute of Biomedical Engineering, University of Toronto, 164 College St., Toronto, ON M5S 3E2, Canada

**Keywords:** smiling, gingiva, dental esthetics, oral surgery, gingivoplasty, Botox

## Abstract

Excessive gingival display (EGD) is defined as more than 2 mm of gingiva display above the maxillary incisors at maximum smile. Various skeletal, dental, and soft tissue etiological factors for EGD have been suggested. This study assessed the effectiveness and stability of surgical (SX) and nonsurgical (NSX) interventions for correction of EGD through a systematic review and meta-analysis following PRISMA 2020 guidelines. An electronic search of Ovid MEDLINE, EMBASE, CENTRAL, Scopus, Web of Science, and LILACS was conducted (2010–2023). Results were expressed as mean change in gingival display using the random-effects model at 1, 3, 6, and 12-month follow-up. At 1 month, SX and NSX treatments yielded a comparable mean reduction of 3.50 mm (2.13–4.86) and 3.43 mm (2.67–4.19) in gingival display, respectively. However, by 6 months, NSX treatments showed a reduction of 0.51 mm compared to 2.86 mm with SX treatments. SX outcomes remained stable past 6 months, while NSX outcomes partially relapsed at 6 months and returned to baseline levels at 12 months. Notably, NSX treatments were more effective in cases with mild initial EGD, while SX treatments showed a better outcome in severe cases. To draw more robust conclusions regarding the treatment outcomes, future primary studies of greater rigor are required.

## 1. Introduction

A smile consists of various elements, including the teeth, the lips, and the appearance of the gingival tissue [1]. The degree of gingival exposure plays a vital role in determining one’s contentment with the esthetics of their smile [2]. Excessive gingival display on smile (EGD) or ‘gummy smile’ is defined as a nonpathological condition that results in disharmony between the maxilla, lips, gingiva, and teeth [1]. There is no agreement about the ideal amount of gingival display; however, Peck and Peck (1995) defined a gingival smile as more than 2 mm of gingiva display above the maxillary central incisors at the maximum smile, which is the limit most commonly used in studies of smile esthetics [3]. Several etiological factors for EGD have been discussed in the literature, including vertical maxillary excess [4], short philtrum height [5], hypermobile upper lip elevator muscles [6,7], altered passive eruption [8], gingival enlargement, retroclination [1] or supra-eruption of maxillary incisors [9]. The recommended treatment modalities for addressing EGD include surgical (SX) and nonsurgical (NSX) treatments. Surgical interventions include orthognathic surgery (with or without V-Y plasty) [5], lip-repositioning surgery (LRS) with or without modifications such as myotomy [10,11], myectomy [12], placement of separator between the lip elevator muscles and nasal spine [13], esthetic crown lengthening (ECL), and gingivectomy. Nonsurgical interventions include injection of botulinum toxin (BTX) and orthodontic intrusion of maxillary teeth [14]. Other uncommonly used surgical or nonsurgical techniques include nasal septum reinforcement using autologous cartilage or an expanded polytetrafluoroethylene implant [15], micro-autologous fat transplantation to the upper lip, nasolabial groove, and ergotrid areas [16], and hyaluronic acid (HA) injection [17,18]. A recent surge in research focused on the treatment of EGD reflects the increasing interest among clinicians and the growing demand from patients to address EGD [19]. There have been systematic reviews conducted on the more common techniques for the reduction of EGD, such as BTX injection [20,21,22,23,24,25,26], LRS [27,28,29,30,31], and the use of skeletal anchorage devices [32]. The majority of studies included in these systematic reviews were case reports and case series with a small patient pool. Consequently, a comprehensive systematic review and meta-analysis of the current literature is required to produce quantifiable results on the outcome and stability of surgical and nonsurgical treatments, identify the limitations, and provide evidence-based indications for clinical practice and insights for future studies [33]. The objective of our systematic review was to appraise the scientific literature, compile the current evidence on outcome and stability of surgical and nonsurgical interventions in adult patients with EGD, and provide evidence-based guidance for clinical practice and insights for future studies.

## 2. Materials and Methods

### 2.1. Protocol

This systematic review adhered to the Preferred Reporting Items for Systematic Reviews and Meta-Analyses (PRISMA) statement guidelines [34]. The protocol for this project was registered in the International Prospective Register of Systematic Reviews (PROSPERO) database under the registration number CRD42022363826.

### 2.2. Information Sources

An electronic search of literature published from 2010 to 2023 was performed on 30 January 2023, in consultation with a health sciences librarian, in six databases: Ovid MEDLINE, EMBASE, Cochrane Central Register of Controlled Trials (CENTRAL), Scopus, Web of Science, LILACS, and the bibliography of relevant studies. All relevant search terms were found by identifying word variants of keywords. Medical Subject Headings (MeSh) were used for the conditions and interventions (Table S1). After finalizing the search syntax for Ovid MEDLINE, it was adapted to other databases.

### 2.3. Eligibility Criteria (Table S2)

- Participants: adult patients 18 years of age or older with a chief complaint of EGD on smiling, with periodontal and systemic health.
- Intervention: surgical and nonsurgical treatments for EGD.
- Comparison: pre-treatment condition.
- Outcome measures: gingival display at maximum smile pre-treatment and at follow-up visits for a minimum of 6 months.
- Study design: randomized controlled trials, non-randomized studies without a control group, prospective, retrospective, comparative cohort, and case–control studies.

Studies with the following criteria were excluded: less than 6 months of follow-up, unpublished and non-peer-reviewed studies, review articles, books, expert opinions, case reports, case series, and clinical guidelines were excluded.

### 2.4. Study Selection

The retrieved studies were screened by two independent reviewers (M.M. and B.H.) using the inclusion and exclusion criteria. The initial screening was conducted by reviewing the title and abstract. Eligible studies were further screened by full-text review and selected for the final analysis. The disagreements were resolved through discussion to reach a consensus.

### 2.5. Data Extraction

Two reviewers (M.M. and B.H.) independently extracted data from the included studies using a pre-prepared data extraction form. The following information was extracted: first author, year of publication, region, study design, participants’ demographics, pre- and post-treatment gingival display, etiology, measurement landmarks, type of intervention, outcome, and follow-up period. Inconsistencies were resolved by discussion between the two reviewers.

### 2.6. Risk of Bias Assessment

The risk of bias for individual studies was assessed using version 2 of the Cochrane risk of bias for randomized trials (RoB 2.0) and risk of bias in non-randomized studies of interventions (ROBINS-1) by two independent reviewers (M.M. and B.H.) [35,36]. Any disagreements were resolved through discussion to reach a consensus.

### 2.7. Data Analysis

The statistical analysis was conducted with assistance from a statistician (A.S.) using the Meta package (version 6.5.0) [37] in R Studio software (version 2023.09.0) [38]. The studies were divided into two groups, surgical and nonsurgical. The surgical studies included LRS with or without modifications, ECL, gingivectomy, GBR, septal cartilage reinforcement, micro-autologous fat transplantation, and V-Y plasty. The nonsurgical studies included BTX injection and orthodontic intrusion. The studies were further subdivided into three groups based the range of initial EGD: mild (2–3.99 mm), moderate (4–5.99 mm), and severe (6 mm or more) [3,39,40,41]. The post-treatment changes in gingival display at four time points (1, 3, 6, and 12 months) were calculated, pooled by using a random-effects model, and expressed as mean difference with a 95% confidence interval (CI) using a single-cohort meta-analysis. When required, the change in the gingival display was calculated by deducting the final gingival margin level from the baseline level.

The sources of heterogeneity were evaluated through a subgroup analysis of surgical and nonsurgical procedures. The studies in each surgical and nonsurgical group were further subdivided according to the study type (randomized or non-randomized) and initial gingival display (mild, moderate, or severe).

## 3. Results

**Figure 1:**
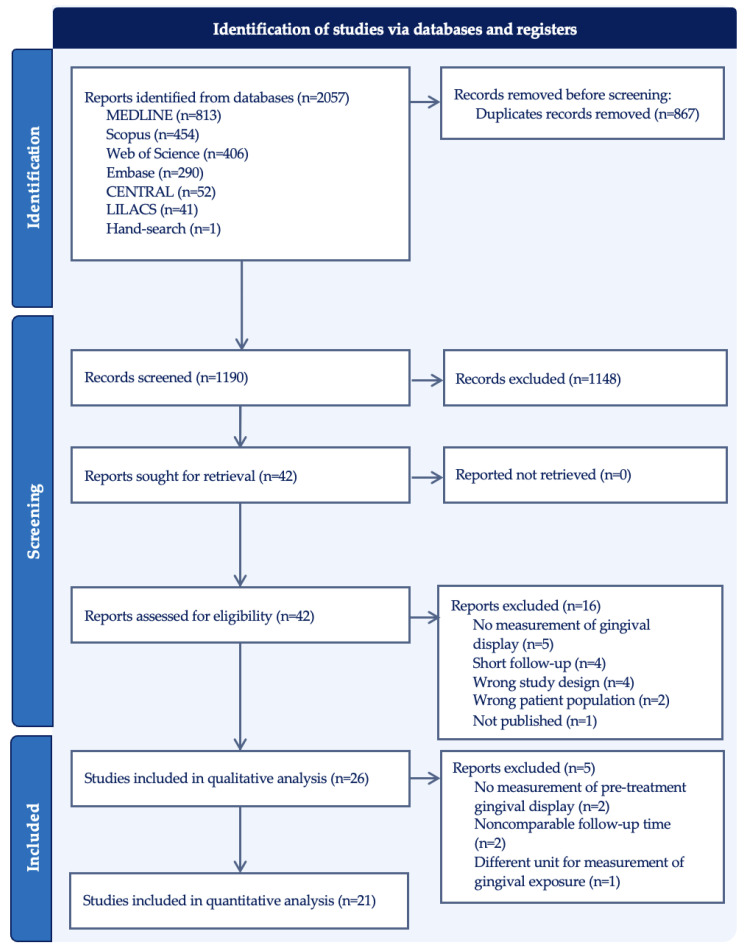
PRISMA flow diagram of identified studies via databases and registers.

The electronic search resulted in 2057 articles for inclusion. Figure 1 outlines the search strategy and the results. After removing the duplicates, 1190 publications were screened by title and abstract for eligibility. A total of 42 articles were included for full-text screening, and 16 articles were excluded due to the following reasons: no measurement of gingival display (n = 5), short follow-up (n = 4), wrong study design (n = 4), wrong patient population (n = 2), or not published (n = 1). A total of 26 studies were included in the data extraction and descriptive analysis, and 21 reports were selected for the meta-analysis. Five articles were excluded from the meta-analysis because the primary outcome could not be compared among them; two did not measure pre-treatment gingival display [42,43], two made measurements at indeterminate timepoints [16,44], and one measured the surface area of gingival exposure [45].

### 3.1. Study Characteristics

The characteristics of the included studies are presented in Table 1. Our systematic review included 20 non-randomized and 6 randomized clinical trials. Only one study declared a conflict of interest due to ownership of a device patent [16]. Of the 687 participants between ages 16 to 60, gender was not specified for 60 patients, and of the remaining 627, 86.5% were female and 13.5% were male. The surgical studies included 323 and the nonsurgical studies included 364 participants.

The etiology of EGD varied among the studies, including hyperactive upper lip (n = 11), short upper lip length (n = 5), altered passive eruption (n = 3), vertical maxillary excess (n = 3), short clinical crown height (n = 1), and nasal septum dysplasia (n = 1). Seven studies excluded VME cases, and six studies did not specify the etiology. Nine studies investigated LRS with different modifications (frenectomy, myotomy, internal dual muscular traction, periosteal suturing, or BTX injection before surgery), two studies used ECL (open-flap, flapless, or laser-assisted), one study investigated gingivectomy with a diode laser, one study used V-Y plasty, one investigated septum cartilage reinforcement, one looked at GBR, one tested fat micro-transplanted into the nasolabial groove, one used orthodontic treatment with extractions and TADs, and ten studies used BTX injection (with different injection sites and dosages and an oral zinc supplement).

The pooled mean pre-treatment gingival display was 5.28 mm, ranging from 2.03 [5] to 7.2 mm [53]. Two studies failed to report the pre-treatment gingival display [42,43]. The shortest follow-up time was 6 months and the longest was 3 years [51]. There were variations among the studies regarding the landmarks used to measure the gingival display.

### 3.2. Risk of Bias

**Figure 2:**
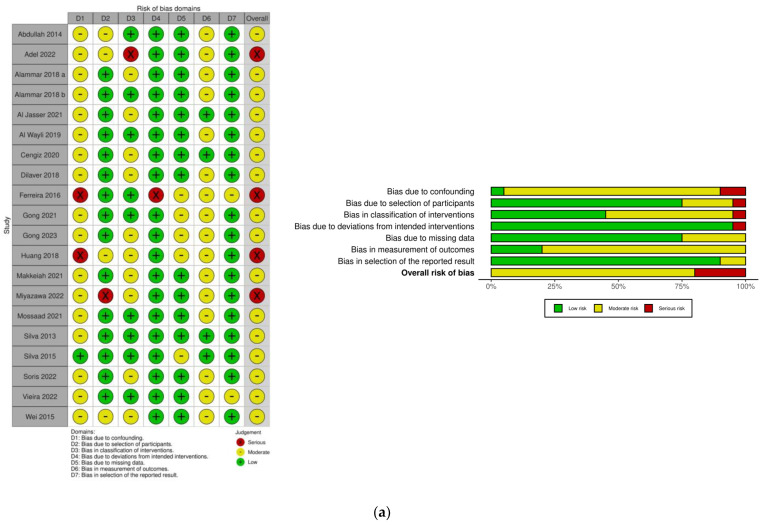

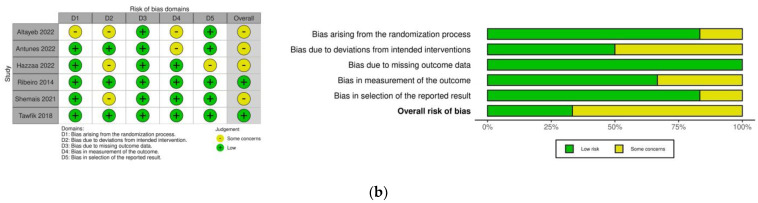
Risk of bias assessment: (**a**) ROBINS-I [5,11,15,39,40,44,45,46,47,48,49,51,53,54,56,57,58,59,60]; (**b**) RoB 2.0. [42,43,50,52,55,61].

The overall risk of bias was graded moderate in 16 (61.5%) and serious in 4 (15.4%) studies in the non-randomized category. Among the randomized studies, the overall risk of bias was graded low in 2 (7.7%) and some concerns in 4 (15.4%) studies (Figure 2).

### 3.3. Primary Outcome

We analyzed the data from 633 patients of whom 228 received surgical treatment, 396 received nonsurgical treatments, and 9 received both surgical and nonsurgical treatments. Of the 21 studies included in the meta-analysis, 10 were surgical, 8 were nonsurgical, and 3 investigated both treatment modalities. The pooled mean pre-treatment gingival display was 5.28 mm with a range of 2.03–7.20 mm (95% CI: 4.46–6.09 mm) (Figure S1).

At 1-month post treatment, the studies with nonsurgical treatment reported a mean reduction of 3.43 mm (95% CI: 2.67–4.19 mm) in gingival display, and the studies with surgical treatment reported a mean reduction of 3.50 mm (95% CI: 2.13–4.86 mm) (Figure S2).

**Figure 3:**
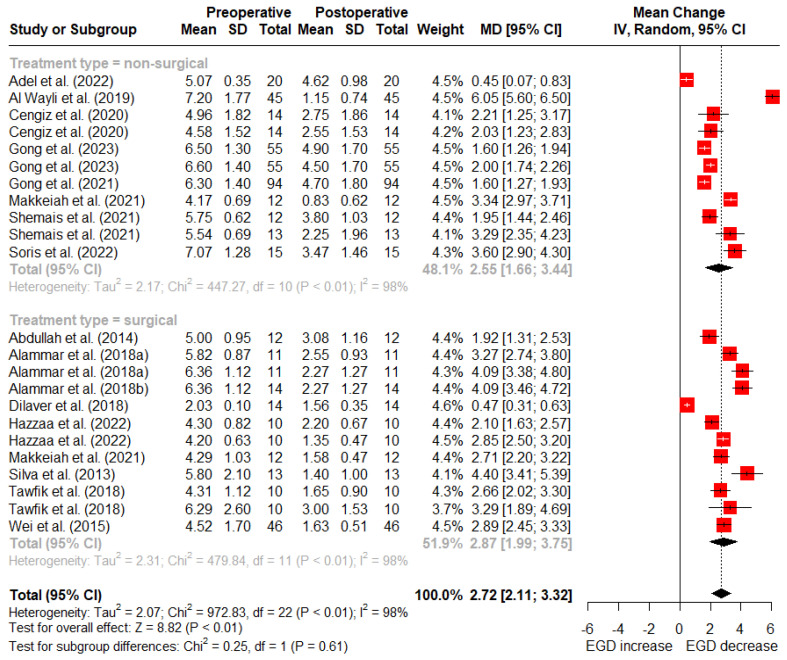
Random-effects meta-analysis of gingival display reduction with surgical vs. nonsurgical treatments at 3-month follow-up (SD: standard deviation; MD: mean difference; CI: confidence interval) [5,11,15,39,40,46,48,49,50,52,53,54,55,56,57,59].

At 3 months post treatment, nonsurgical treatments showed a mean reduction of 2.55 mm (95% CI: 1.66–3.44 mm) in gingival display, while surgical treatments showed an average reduction of 2.87 mm (95% CI: 1.99–3.75 mm). No statistically significant difference was observed between the surgical and nonsurgical treatments at 1- and 3-month follow-up (*p* = 0.93 and *p* = 0.61) (Figure 3).

**Figure 4:**
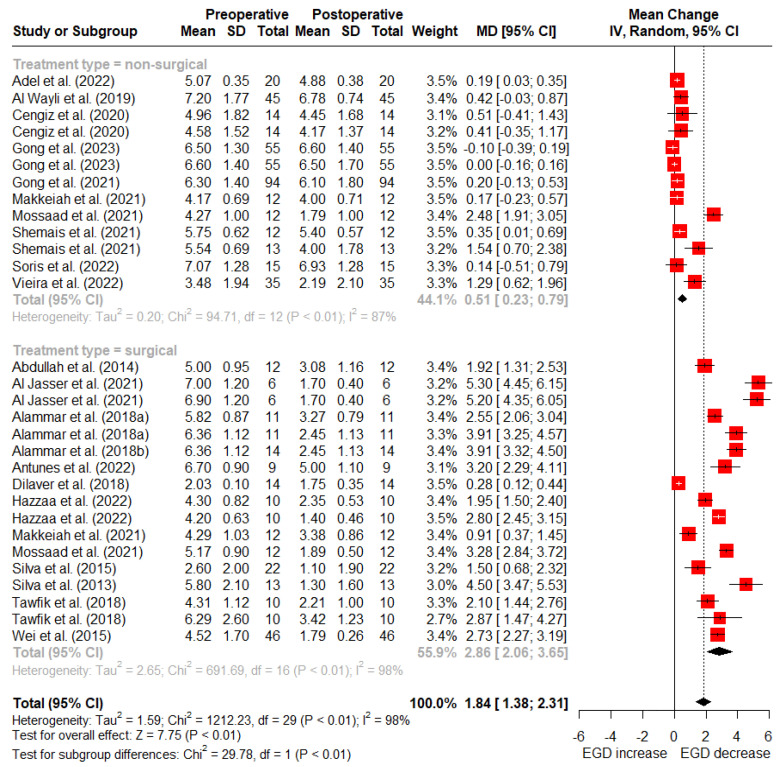
Random-effects meta-analysis of reduction in gingival display with surgical vs. nonsurgical treatments at 6-month follow-up (SD: standard deviation; MD: mean difference; CI: confidence interval) [5,11,15,39,40,46,47,48,49,50,51,52,53,54,55,56,57,58,59,60,61].

At 6 months post-treatment, nonsurgical treatments reported a mean reduction of 0.51 mm (95% CI: 0.23–0.79 mm) in gingival display, whereas surgical treatments reported an average reduction of 2.86 mm (95% CI: 2.06–3.65 mm) (Figure 4).

**Figure 5:**
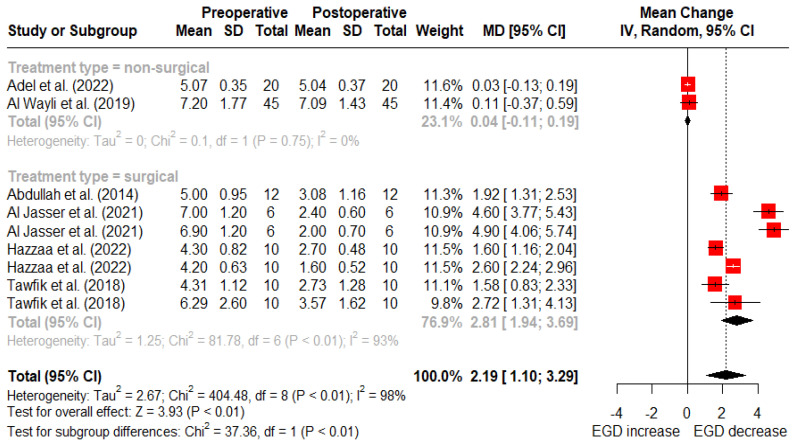
Random-effects meta-analysis of gingival display reduction with surgical vs. nonsurgical treatments at 12-month follow-up (SD: standard deviation; MD: mean difference; CI: confidence interval) [11,50,51,52,53,56].

At 12 months post-treatment, nonsurgical treatments reported an average reduction of 0.04 mm (95% CI: −0.11–0.19 mm) in gingival display, while surgical treatments reported a mean reduction of 2.81 mm (95% CI: 1.94–3.69 mm) (Figure 5). There was a statistically significant difference between surgical and nonsurgical treatment types at 6- and 12-month follow-up (*p* < 0.01).

### 3.4. Subgroup Analysis

A subgroup analysis was performed by comparing the randomized and non-randomized surgical treatments at 6 months follow-up. The results showed that the treatment effect was only slightly higher in the non-randomized studies (2.98 mm) compared to the randomized studies (2.50 mm) and the difference was not statistically significant (*p* = 0.43) (Figure S3). Similarly, the treatment effects at 6 months were comparable between randomized and non-randomized nonsurgical treatments (0.88 and 0.46 mm, respectively) with no statistically significant difference (*p* = 0.50) (Figure S4).

The studies were categorized based on the pre-treatment gingival display as mild (2–3.99 mm), moderate (4–5.99 mm), and severe (6mm or more) EGD. The random-effects analysis for the surgical treatments at 6 months showed that the treatment effect in severe EGD (4.12 mm) was significantly larger than in moderate (2.48 mm) or mild (0.82 mm) EGD (*p* < 0.01) (Figure S5). The random-effects analysis for the nonsurgical treatments at 6 months follow-up showed that the treatment effect was smallest in the studies with severe initial gingival display (0.06 mm) compared to moderate (0.78 mm) or mild (1.29 mm) (*p* < 0.01) (Figure S6).

## 4. Discussion

In this investigation, the systematic review and meta-analysis included 26 and 21 reports, respectively, published between 2013 and 2023. A total of 633 patients were pooled, of which 228 underwent surgical treatment, 396 nonsurgical, and 9 had a combination of both (botox and LRS). The results of the meta-analysis suggested that there was no significant difference between the two treatment modalities at 1 and 3 months post treatment. The surgical outcomes remained stable at 6 and 12 months, while nonsurgical outcomes partially relapsed at 6 months and returned to baseline at 12 months. The initial severity of gingival exposure and the treatment modality played a role in the treatment effects. Gong et al. (2023) categorized the pre-treatment gingival show into three groups based on severity: mild (3–5 mm), moderate (5–7 mm), and severe (greater than 7 mm) [39]. In this meta-analysis, the studies were divided into three groups based the range of initial EGD: mild (2–3.99 mm), moderate (4–5.99 mm), and severe (6 mm or more). At 6 months, surgical treatments showed significantly greater reduction in gingival display in severe compared to moderate or mild EGD. On the other hand, the treatment effect of nonsurgical treatments was smaller in studies with severe initial gingival display compared to moderate or mild.

### 4.1. Lip-Repositioning Surgery

There are several variations for LRS with the goal of relapse minimization and improvement of long-term stability by preventing reinsertion of smile muscles to their original position [51]. These variations include frenectomy, use of adjuvants (botox), muscular amputation (myotomy), muscle dissection, muscle containment with insertion of polyester sutures [62], and periosteal suturing [27]. Mendoza-Geng et al. (2022) suggested that the use of periosteal suturing with LRS caused the greatest decrease in EGD, with 5.22 mm (4.23–6.21) at 6 months and 4.94 mm (3.86–6.02) at 12 months post treatment [27]. Similarly, Dos Santo-Pereira et al. (2020) reported a reduction of 2.87 mm (1.91–3.82) after 3 months, which decreased to 2.71 mm (1.95–3.47) at 6 months and 2.10 mm (1.48–2.72) at 12 months, showing a relapse rate of 25% after 12 months [29]. Younespour et al. (2021) showed a reduction range of 2.68–3.22 mm with various LRS modalities [28]. Descriptively, the greatest gingival display reduction was associated with the modality that did not include frenectomy or myotomy [28].

Long-term stability might be one of the most controversial aspects of LRS. Alammar and Heshmeh (2018) explained that relapse may occur due to incomplete stripping of the muscles from the bone during the surgical procedure or muscle memory reattachment to the previous pre-bone base [49]. Nonetheless, due to the lack of follow-ups longer than 12 months, it is not possible to confirm if this decrease continues to the baseline levels.

### 4.2. Gingivectomy and Crown Lengthening

In cases where there is an excessive amount of keratinized gingiva with short and square-shaped teeth with sufficient distance between the cementoenamel junction (CEJ) and the osseous crest, gingivectomy is indicated [63]. If the osseous crest is found to be close to the level of the CEJ, osseous resection with flap elevation may be required [63]. Where there are clinically visible short teeth with a limited amount of keratinized gingival tissue, treatment involves apical repositioning of the entire dento-gingival complex with or without osseous reduction [63].

Diode 655–980 nm lasers have been used for gingivectomy procedures in patients with EGD [60]. It prevents bleeding by sealing the blood vessels and inhibits pain receptors, contributing to reduced discomfort during the procedure [64]. One article studied the effect of gingivectomy and ostectomy (open-flap and flapless) performed with an Er,Cr–YSGG laser [43]. The outcome measure used was the gingival margin level, and no difference was found between the two techniques.

### 4.3. V-Y Plasty

V-Y plasty is a method used to cover the increased gingival display that is expected after Le Fort I osteotomy [5]. Dilaver and Uckan (2018) found that the benefits of V–Y plasty following Le Fort I are greater than those of the V-Y plasty applied as a standalone procedure [5]. Muradin et al. (2009) employed a modified alar cinch suture technique, which involved passing sutures through the levator and nasal muscles, including the periosteum, and threading them through the nasal septum [65]. This was combined with a muco-musculo-periosteal V-Y closure following Le Fort 1 osteotomy [65]. Postoperatively, there appeared to be a reduction in the vertical mobility of the corners of the mouth when observing the maximum smile, which could potentially contribute to improving excessive gingival display [65]. Due to the limited number of studies, a conclusion regarding the effect of Le Fort 1 impaction or V-Y plasty on gingival display cannot be drawn.

### 4.4. Guided Bone Regenration

Guided bone regeneration was suggested by one study as a camouflage treatment for VME [45]. This technique showed an improvement in EGD of 40.7% when performed alone and 60% when combined with a crown-lengthening procedure [45].

### 4.5. Septum Cartilage Reinforcement

Wei et al. (2015) investigated the role of nasal septal dysplasia in the development of EGD among the Asian populations [15]. The absence of natural antagonism due to nasal septal cartilage dysplasia leads to an upward movement of the upper lip during smiling, resulting in excessive gingival exposure [15]. The outcome showed that there was an average 3.34 mm reduction in EGD at 1 month with an average minimal relapse of 0.61 mm at 6 months post treatment [15]. This was a retrospective study with variable follow-up time among cases.

### 4.6. Micro-Autologous Fat Transplantation

In a study by Huang et al. (2018), MAFT in the nasolabial groove, ergotrid, and upper lip was used to camouflage EGD by blocking the upper lip elevator muscles and increasing the vertical height of the upper lip [16]. Due to the paucity of evidence on MAFT, there remains a gap in our understanding of the long-term outcomes associated with these procedures [16].

### 4.7. Botulinum Toxin

#### 4.7.1. Injection Site

The levator labii superioris (LLS), levator labii superioris alaeque nasi (LLSAN), and zygomaticus minor (Zmi) muscles determine the degree of upper lip elevation and converge near a triangular region in the nasolabial fold [66]. The center of this triangle, Yonsei point, has been suggested because the optimal injection site could encompass all three muscles with a single injection [67]. Cengiz et al. (2020) suggested isolated injection into the orbicularis oris (OO) at the junction of the elevator muscles. However, the treatments results with this approach were inferior to those of isolated LLSAN injection (53% versus 61% reduction in gingival exposure) [54]. The OO muscle is involved in many basic facial expressions; therefore, the discomfort associated with the injection, subsequent muscle weakness, or paralysis of this muscle should be considered [54]. Razmaitė and Trakinienė (2021) stated that the different techniques of BTX administration and injection sites did not significantly affect the clinical outcome [25]. A systematic review by Lam and Chan (2022) confirmed that there was no correlation between the number of injection sites and improvement in EGD [21].

#### 4.7.2. Dosage and Initial Severity of EGD

In a study by Gong et al. (2021), it was stated that the effectiveness of an average dose of BTX was influenced by the severity of EGD and the patient’s gender rather than the underlying etiology [40]. For female patients with a baseline anterior gingival exposure of 6 mm or more (or male patients), the BTX dose could be proportionally increased [40]. Additionally, Andriola et al. (2021) confirmed that the initial amount of EGD is an important factor in BTX efficiency [41].

#### 4.7.3. Stability

A systematic review by Chagas et al. (2018) found that the gingival display was considerably reduced at 2 weeks post treatment (4.05 mm) and remained stable until 8 weeks post treatment [25]. Another systematic review by Nasr et al. (2016) showed that the results of BTX lasted within a range of 12 to 24 weeks [22]. Rasteau et al. (2022) found similar results that the improvement in gingival display persisted for 12 to 36 weeks [20]. A meta-analysis by Zengiski et al. (2022) showed a similar decrease in gingival display with a slight decrease in effect size at 12 weeks [23]. After 24 weeks, despite the statistical significance, the observed effect size was very small approaching the initial values, and thus it was not clinically significant [23]. Lam and Chan (2022) confirmed that the results of injection started to disappear after 12 weeks, and normal function returned at 24–30 weeks [21]. As a zinc-dependent metalloprotease, BTX exerts its muscle-paralyzing effect in the presence of a zinc molecule [68]. The clinical efficacy and duration of Botox A injections can fluctuate based on the zinc levels in the body [68]. Shemais et al. (2021) showed that zinc supplementation prolonged the duration of BTX’s effect for more than 6 months. A limitation of this study was the lack of a placebo supplement for the control group [55].

### 4.8. Orthodontic Treatment

The use of miniscrews offers anchorage for intrusion of anterior teeth in individuals with EGD and deep overbites. Miyazawa et al. (2022) found that orthodontic treatment combined with midpalatal miniscrews can be an effective alternative to orthognathic surgery [44]. However, the degree of lip incompetence, length, and mobility of the upper lip were not assessed in this study [44]. A systematic review by Alshammery et al. (2021) found TSADs to be useful in the correction of a deep bite [32]. The reported dentoalveolar intrusion was 2.25–2.9 mm; however, no soft tissue measurement of the gingival display was reported [32].

### 4.9. Limtations of the Current Study

The main limitation of our systematic review was the high level of heterogeneity. The ideal study design for a systematic review is a randomized clinical trial; however, our review was based on a sample of mainly non-randomized studies. It has been suggested that well-conducted prospective non-randomized studies can provide complementary evidence [69]. The variable etiology of EGD (and, in some studies, lack of identification of etiology) and the variability of landmarks for the measurement of gingival display can affect the treatment outcome. Furthermore, the reproducibility of maximum smile before and after treatment was not validated. Additionally, the age range of the study participants was wide. It has been shown that the severity of EGD can change with age due to the sagging of perioral soft tissues [70]. The variations in surgical techniques and injection sites and dosages for BTX injection is an important limitation when determining the treatment technique with the best outcome. The aforementioned limitations restrict the generalizability and applicability of the results.

### 4.10. Future Directions

While it is imperative to regard the results of this meta-analysis with caution due to the high heterogeneity and the aforementioned contributing factors, this review may serve as a starting point for future research endeavors featuring more rigorous research designs. To draw more robust conclusions regarding the short- and long-term treatment outcomes of EGD correction, future primary studies of greater rigor are needed. The studies should adopt a randomized and controlled design and include a detailed diagnosis of EGD etiology, patient characteristics, and quantitative results. In cases in which randomization is not easily employed, future studies should, as a minimum, ensure that the groups compared are matched.

## 5. Conclusions

This systematic review suggests that both surgical and nonsurgical approaches can successfully address EGD. Surgical treatments seem to be more effective for severe EGD and last up to 12 months, while nonsurgical treatments seem to be more effective for mild EGD with a tendency to relapse after 6 months. Given the limitations of this systematic review including the heterogeneity among included studies, the variability in measurement landmarks for gingival display, the broad age range of participants, and inclusion of randomized and non-randomized studies, the findings of this synthesis must be interpreted with caution. To draw more robust conclusions regarding the short- and long-term treatment outcomes of EGD correction and generalize the findings to various patient populations and different ages, future primary studies of greater rigor are needed. Additionally, management of EGD and selection of appropriate treatment options should be approached with consideration of the underlying etiology, patient education regarding the short- and long-term effects of surgical and nonsurgical treatments, and informed consent. As previously cited in the literature [19], each treatment modality is tailored to address a specific cause of EGD. Orthognathic intervention is recommended for skeletal etiology, while soft tissue etiologies warrant treatments targeted at soft tissues, and dental causes should be addressed accordingly. In certain scenarios, clinicians may encounter patients who decline a recommended procedure due to its invasive nature. Hence, clinicians should be equipped with knowledge and preparedness to suggest alternative treatment options in such instances.

## Figures and Tables

**Table 1:** Study characteristics.

Author/ Year	Country	Study Design	Population Age N (F:M)	Etiology	Intervention Number (n)	Comparison Number (n)	Landmark for Measurement	Pre-Treatment Gingival Exposure	Post-Treatment Gingival Exposure	Follow-Up	Measured Stability at Follow-Up
**Silva 2013** [46]	**Brazil**	prospective	28.7 ± 11.0 (19–49) N = 13 (11:2)	hyperactive upper lip (>8 mm mobility)	modified LRS without excision of maxillary midline labial frenum	baseline	from the inferior border of the upper lip vermillion to the GM of the right central incisor	5.80 ± 2.1	3 months 1.40 ± 1.00	3, 6 months	6 months 1.30 ± 1.60
**Abdullah 2014** [11]	**Saudi Arabia**	N/A	23.75 ± 2.89 (20–29) N = 12 (10:2)	not specified (acceptable vertical dimension and tooth–gingiva relationship)	LRS with frenectomy and myotomy of LLS muscles using circumdental sutures	baseline	N/A	5.0 ± 0.95	1 month 2.66 ± 0.77	1, 3, 6, 12 months	3, 6, 12 months 3.08 ± 1.16
**Ribeiro 2014** [42]	**Brazil**	split-mouth randomized controlled trial	27.5± 5.8 (21–40) N = 28 (20:8)	APE in at least three maxillary teeth (excluded: orthodontic treatment)	conventional open-flap (OF) ECL (n = 28)	flapless (FL) ECL (n = 28)	relative GM on buccal surface measured using a surgical stent	N/A	reduction from baseline OF: 1.3 ± 0.5 FL: 1.1 ± 0.5	3, 6, 12 months	reduction at 3 months: OF: 1.2 ± 0.5 FL: 1.0 ± 0.5 reduction at 6 and 12 months: OF and FL: 1.0 ± 0.5
**Silva 2015** [47]	**Brazil**	prospective	23.5 ± 2.7 (19–32) N = 32 (26:6)	APE—width/length ratio ≥0.85 and gingival margin incisal to tooth cervical convexity (excluded: orthodontic treatment, heavy restorations)	ECL with osteoplasty and osteotomy	baseline	distance between central incisor GM during active smile and the inferior border of the upper lip vermilion	2.6 ± 2.0	N/A	6 months	1.1 ± 1.9
**Wei 2015** [15]	**China**	prospective	28.7 ± 7.7 (18–49) N = 46 (gender N/A)	nasal septum dysplasia with increased columella upward maximum movability (CUMM)	septum cartilage reinforcement and additional extension as needed (auricular cartilage n = 18, costicartilage n= 13, ePTFE implant n = 15, augmentation rhinoplasty n = 31)	150 volunteers	N/A	4.52 ± 1.7	1 month 1.18 ± 0.59	1, 3, 6 months	3 months 1.63 ± 0.51 6 months 1.79 ± 0.26
**Ferreira 2016** [45]	**Brazil**	prospective	26 (20–49) N = 12 (F)	VME	GBR with xenogenic bone substitute (Bio-OssTM) and resorbable membrane (Bio-GideTM); n = 8 required ECL	baseline	measured surface area of gingival exposure	275.44 mm^2^ (≥5 mm during full posed smile)	N/A	1, 6, 12 months	12 months no ECL: reduction of 112.01 mm^2^ with ECL: reduction of 167.01 mm^2^
**Alammar 2018 a** [48]	**Syria**	prospective	18–38 N = 22 (19:3)	short upper lip, hyperactive lip elevator muscles (>8 mm mobility)	conventional LRS (partial-thickness flap excluding myotomy elevator muscles) (n = 11)	modified LRS (full-thickness flap with myotomy of elevator muscles) (n = 11)	inferior border of upper lip vermillion to the gingival margin of the anterior maxillary teeth	conventional: 5.82 ± 0.87 modified: 6.36 ± 1.12	1 month conventional: 2.18 ± 0.75 modified: 0.91 ± 1.22	1, 3, 6 months	3 months conventional: 2.55 ± 0.93 modified: 2.27 ± 1.27 6 months conventional: 3.27 ± 0.79 modified: 2.45 ± 1.13
**Alammar 2018 b** [49]	**Syria**	prospective	18–38 N = 14 (gender N/A)	short upper lip, hyperactive lip elevator muscles (>8 mm mobility) (excluded: VME, gingival display >6 mm)	LRS (full-thickness flap, V-shaped in the upper lip frenum to preserve the labial midline) with myotomy of the elevator muscles.	Baseline	inferior border of the upper lip vermillion to the gingival margin of the anterior maxillary teeth during full active smile	6.36 ± 1.12	1 month 0.91 ± 1.22	1, 3, 6 months	3 months 2.27 ± 1.27 6 months 2.45 ± 1.13
**Dilaver 2018** [5]	**Turkey**	prospective	23.2 (18–30) N = 14 (12:2)	short upper lip (<20 mm in F, <23 mm in M), VME cases that refused surgery (excluded: gingival hyperplasia, APE, short clinical crown, prior V–Y plasty)	V-Y plasty with supra-periosteal dissection of submucosa and underlying muscles	baseline	middle uppermost point of the gingival margin of each crown and the corresponding locations on the upper lip measured for central incisors	2.03 ± 0.1	1 month 0.80 ± 0.12	1, 3, 6 months	3 months 1.56 ± 0.35 6 months 1.75 ± 0.35
**Tawfik 2018** [50]	**Egypt**	single-blinded, randomized clinical trial	≥18 N = 20 (18:2)	not specified (all with normal clinical crown dimensions)	LRS with myotomy (lower incision like Rosenblatt and Simon and upper incision like Rubenstein and Kostianovsky modification) (n = 10)	conventional LRS without myotomy (n = 10)	N/A	with myotomy: 6.29 ± 2.6 without myotomy: 4.31 ± 1.12	3 months with myotomy: 3.00 ± 1.53 without myotomy: 1.65 ± 0.9	3, 6, 12 months	6 months with myotomy: 3.42 ± 1.23 without myotomy: 2.21 ± 1.0 12 months with myotomy: 3.57 ± 1.62 without myotomy: 2.73 ± 1.28
**Al Jasser 2021** [51]	**Saudi Arabia**	prospective	≥18 years N = 12 (twins) (F)	Excluded: VME, skeletal deformity, previous Botox or fillers, short upper lip, APE	LRS by LipStaT technique—no suture in the thick connective tissue (n = 6)	LRS with deep periosteal suture in the thick connective tissue (n = 6)	molar to molar at 3 buccal locations, from gingival margin to base of the upper lip during full dynamic smile	without periosteal suture: 7.0 ± 1.2 with periosteal suture: 6.9 ± 1.2	6 months without periosteal suture: 1.7 ± 0.4 with periosteal suture: 1.7 ± 0.4	1, 2, 3, 4 weeks, 1, 6 months, 1, 2, 3 years	1 and 2 years without periosteal suture: 2.4 ± 0.6 with periosteal suture: 2 ± 0.7 3 years without periosteal suture: 5.0 ± 1.8 with periosteal suture: 3.5 ± 1.4
**Altayeb 2022** [43]	**Qatar-USA**	randomized clinical study	22–45 N = 36 (22:14)	APE (gingival overlap of over 19% of the anatomical crown height)	Er,Cr:YSGG (2780 nm) laser–assistedopen-flap (OF) ECL with variable-thickness flap (n = 18)	Er,Cr:YSGG (2780 nm) laser–assisted flapless (FL) ECL (n = 18)	GM level of central incisors (from incisal edge to gingival zenith)	N/A	reduction from baseline OF: 2.37 ± 0.54 FL: 2.28 ± 0.39	1, 3, 9 months	Reduction at 1 month OF: 2.34 ± 0.48 FL: 2 ± 0.48 3 months OF: 2.16 ± 0.48 FL: 1.9 ± 0.33 9 months OF: 2.09 ± 0.49 FL: 2.01 ± 0.41
**Hazzaa 2022** [52]	**Egypt**	randomized clinical trial	iMTA: 30.7 ± 5.0 LRS alone: 29.9 ± 3.1 N = 20 (14:6)	excluded: VME >8 mm	modified LRS with internal dual muscular traction approach (iMTA) and evelo-periosteal suturing (n = 10)	modified LRS (excluding maxillary labial frenum) (n = 10)	gingival margin until the upper border of the lip	iMTA: 4.2 ± 0.63 LRS alone: 4.3 ± 0.82	3 months iMTA: 1.35 ± 0.47 LRS alone: 2.2 ± 0.67	3, 6, 12 months	6 months iMTA: 1.4 ± 0.46 LRS alone: 2.35 ± 0.53 12 months iMTA: 1.6 ± 0.52 LRS alone: 2.7 ± 0.48
**Huang 2018** [16]	**Taiwan**	N/A	23–40 N = 7 (6:1)	N/A	fat micro-transplantation into the nasolabial groove, ergotrid, and upper lip using MAFT-GUN (mean volume: 16.1 mL)	baseline	gum line at the midline of the incisors (right and left) and canines (right and left) to the lowest portion of the upper lip	4.4 ± 2.5	measured at follow-up	6–24 months	−0.5 ± 1.2
**Al Wayli 2019** [53]	**Saudi Arabia**	prospective	30.5 ± 9.43 (18–45) N = 45 (F)	hyperfunctional upper lip elevator muscles; other diagnosed etiologies were corrected before the study (excluded VME)	BTX, Dysport dilution: 100 units/2.0 mL injection site: Yonsei points bilaterally dose: 3 units/side	baseline	distance between the lowest margin of the upper lipperpendicular to the midportion of the maxillary central incisor’s gingival margin	7.20 ± 1.77	12 weeks 1.15 ± 0.74	2, 12, 24, 36 weeks	24 weeks 6.78 ± 0.74 36 weeks 7.09 ± 1.43
**Cengiz 2020** [54]	**Turkey**	prospective cohort	22.11 ± 4.55 (19.8–38.1) N = 28 (21:7)	not specified (excluded: >8 mm EGD)	BTX, Allergan dilution: 100 units/2.0 mL injection site: LLSAN on the most superior point of the nasolabial fold dose: 2.5 units/side	BTX, Allergan dilution: 100 units/2.0 mL injection site: OO, 5 mm inferior to the central and most inferior point of the nostrils dose: 1.25 units/side	distance between the gingival margin on the midline of the maxillary right central incisor and the inferior border of the upper lip	LLSAN: 4.96 ± 1.82 OO: 4.58 ± 1.52	3 days LLSAN: 2.48 ± 1.85 OO: 2.80 ± 1.75	3, 15 days, 1, 4, 5, 6 months	15 days LLSAN: 1.92 ± 1.70 OO: 2.16 ± 1.65 1 month LLSAN: 1.83 ± 1.74 OO: 2.2 ± 1.57 4 months LLSAN: 2.75 ± 1.86 OO: 2.55 ± 1.53 6 months LLSAN: 4.45 ± 1.68 OO: 4.17 ± 1.37
**Gong 2021** [40]	**China**	prospective	27 (25–32) N = 94 (77:17)	excluded: previous disease or treatment affecting the position of the gingiva or upper lip	BTX, Allergan dilution: 100 units/2.0 mL injection site: LLSAN at the muscle bulge at the uppermost part of the nasolabial fold dose: 2 units/side	baseline	distance between the inferior margin point of the right central incisor and the lower margin of the upper lip	6.3 ± 1.4 (anterior)	4 weeks 3.9 ± 2.0	4, 12, 32 weeks	12 weeks 4.7 ± 1.8 32 weeks 6.1 ± 1.8
**Shemais 2021** [55]	**Egypt**	randomized controlled clinical trial	25 ± 4 (20–30) N = 25 (23:2)	hypermobile and short lip, VME of maximum 4 mm, normal clinical crown dimensions	50 mg oral zinc supplement for 4 days prior to injection with BTX-A, Allergan dilution: N/A injection site: Yonsei points (1 cm lateral to the ala and 3 cm above the lip line) dose: 3 units/side (n = 13)	BTX-A, Allergan without zinc supplement (n = 12)	recorded using a UNC15 periodontal probe at the midpoint of the right central incisor and premolars at maximum smile	Zinc and BTX: 5.54 ± 0.69 BTX only: 5.75 ± 0.62	2 weeks Zinc and BTX: 0.65 ± 1.26 BTX only: 1.41 ± 0.86	2, 6, 12, 18, 24 weeks	12 week Zinc and BTX: 2.25 ±1.06 BTX only: 3.8 ± 1.03 24 weeks Zinc and BTX: 4 ± 1.78 BTX only: 5.4 ± 0.57
**Adel 2022** [56]	**Egypt**	N/A	25–45 N = 20 (F)	hypermobile upper lip (twice normal translation range of 6–8 mm)	BTX dilution: 100 unit/2 mL injection site: Yonsei point for all cases, additional 2 points at insertion of ZM muscles for mixed smile cases dose: 1 unit at Yonsei point per 1 mm of gingival show, 0.5 unit for other points; additional injection at 4 and 8 months	baseline	vertical distance from free gingival margin of central incisor to lower border of upper lip measured digitally on smiling	5.07 ± 0.35	14 days 0 ± 0	14 days, 4, 8, 12 months	4 months 4.62 ± 0.98 8 months 4.88 ± 0.38 12 months 5.04 ± 0.37
**Miyazawa 2022** [44]	**Japan**	retrospective	23.2 ± 4.2 (17–33) N = 16 (F)	gummy smile ≥3.0 mm, maxillary or bimaxillary protrusion	orthodontic treatment with extraction of 4 premolars and two self-drilling miniscrews in the midpalatal suture ligated to a modified transpalatal arch	baseline	movement of prosthion was used to quantify the changes in gingival exposure at the maxillary central incisors	4.6 ± 1.2	1.6 ± 1.6	average treatment duration: 4 years 2 months (2.5–8 years)	N/A
**Soris 2022** [57]	**India**	N/A	18–40 N = 15 (10:5)	hyperactive lip elevator muscles or VME (excluded: short upper lip)	BTX, Allergan dilution: 4 unit/0.1 mL injection site: Yonsei points and upper lip philtrum dose: 4 units/side at Yonsei points and 2 units at upper lip philtrum	baseline	crest of the gingiva (centrals in anterior and premolars in posterior gummy smile) to the lower most border of the upper lip	7.07 ± 1.28	7 days 5.07 ± 0.96	3, 7, 15 days, 1, 2, 3, 4, 5, 6 months	15 days 3.40 ± 1.06 1 month 3.07 ± 1.33 3 months 3.47 ± 1.46 6 months 6.93 ± 1.28
**Vieira 2022** [58]	**Brazil**	prospective	25.5 ± 5.6 N = 35 (30:5)	muscular hyperfunction (excluded: VME, >5 mm of gingival display)	BTX, Dysport dilution: 2 units/0.01 mL injection site: LLSAN muscle dose: 2 units/side	baseline	linear distance between lower margin of the upper lip to the incisal edge of the maxillary central incisor minus the size of the crown of the right maxillary central incisor	3.48 ± 1.94	2 weeks −0.04 ± 1.57	2, 32 weeks	32 weeks 2.19 ± 2.10
**Gong 2023** [39]	**China**	prospective	28.9 ± 5.9 (18–60) N = 55 (51:4)	hypermobility of upper lip, short clinical crown, dentoalveolar extrusion, short upper lip (excluded: history of or active orthodontic treatment)	BTX, Allergan simplified injection dilution: 100 units/2.5 mL injection site: bilateral LLSAN dose: 2 units/side	individualized injection after 8 months dilution: 100 units/2.5 mL injection site: bilateral LLSAN and Yonsei points dose: base on severity of anterior gingival smile: mild (3–5 mm) 2 units/side moderate (5–7 mm): 3 units/side severe (≥7 mm): 5 units/side	distance between the superior margin of the right incisor and the lower margin of the upper lip	simplified: 6.5 ± 1.3 individualized: 6.6 ± 1.4	4 weeks simplified: 4.1 ± 1.8 individualized: 3.8 ± 1.6	4, 12, 32 weeks	12 weeks simplified: 4.9 ± 1.7 individualized: 4.5 ± 1.7 32 weeks simplified: 6.6 ± 1.4 individualized: 6.5 ± 1.7
**Makkeiah 2021** [59]	**Syria**	prospective cohort	18–41 N = 24 (F)	hyperactive upper lip	LRS (partial-thickness flap) (n = 12)	BTX A dilution: 100 units/2 mL injection sites: 3 sites bilaterally into the LLS and LLSAN muscles dose: 4–6 units/site (n = 12)	difference between the lower margin of the upper lip and the superior margin of the right and left central incisors and canines at maximum smile	LRS: 4.25 ± 0.85 BTX: 4.29 ± 0.53	2 weeks LRS: 1.35 ± 0.72 BTX: 0.46 ± 0.38	2 weeks, 2, 6 months	2 months LRS: 2.17 ± 0.52 BTX: 1.73 ± 0.38 6 months LRS: 3.23 ± 0.74 BTX: 3.94 ± 0.55
**Mossaad 2021** [60]	**Egypt**	comparative cohort	25–35 N = 24 (F)	excluded: VME	diode laser gingivectomy (premolar to premolar) (n = 12)	BTX, Allergan: dilution: 100 units/2.5 mL injection site: 4 units on both sides of the nasolabial fold at Yonsei points and 2 units below the nose (orbicularis oris muscle) dose: 6 units/side (n = 12)	vertical exposure of gingiva from the lower border of the upper lip to the free gingival margin of the maxillary anterior teeth	laser: 5.17 ± 0.9 BTX: 4.27 ± 1.0	1 week laser: 1.89 ± 0.5 BTX: 1.79 ± 1.0	1 week, 1, 3, 6 months	at 6 months, BTX group returned to pre-treatment evel but effect of laser remained; no measurements reported
**Antunes 2022** [61]	**Brazil**	randomized clinical trial	20–46 N = 18 (F)	hyperactive upper lip (lip displacement ≥ 9 mm) with or without other causes of EGD such as VME or APE (excluded: short lip)	BTX A, Allergan 15 days beforeLRS (partial-thickness flap) dilution: 50 units/1 mL injection site: bilateral LLSAN, LLS, Zmi muscles injection dose: 2 units/muscle, 6 units/side (n = 9)	LRS (partial-thickness flap) alone (n = 9)	cervical margin of the maxillary right central incisor to the lip at maximum smile	BTX and LRS: 6.1 ± 0.8 LRS: 6.7 ± 0.9 mm	N/A	15 days, 3, 6 months	6 months BTX and LRS: 1.6 ± 1.4 LRS: 5.0 ± 1.1

APE: altered passive eruption; BTX: botulinum toxin; ECL: esthetic crown lengthening; EGD: excessive gingival display; FL: flapless; GM: gingival margin; LLS: levator labii superioris muscle; LLSAN: levator labii superioris alaeque nasi muscle; N/A: not available; OF: open flap; OO: orbicularis oris muscle; VME: vertical maxillary excess; ZM: zygomaticus major muscle; Zmi: zygomaticus minor muscle.

## Data Availability

The original contributions presented in the study are included in the article/Supplementary Materials, further inquiries can be directed to the corresponding author/s.

## Supplementary Materials

The following supporting information can be downloaded at: https://www.mdpi.com/article/10.3390/dj12060154/s1, Table S1: Search keywords, synonyms, and related concepts; Table S2: Inclusion and exclusion criteria; Figure S1. Random-effects meta-analysis of pre-treatment gingival display; Figure S2. Random-effects meta-analysis of gingival display reduction with surgical vs. nonsurgical treatments at 1-month follow-up; Figure S3. Random-effects meta-analysis of gingival display reduction with randomized vs. non-randomized surgical treatments at 6-month follow-up; Figure S4. Random-effects meta-analysis of gingival display reduction with randomized vs. non-randomized nonsurgical treatments at 6-month follow-up; Figure S5. Random-effects subgroup analysis of gingival display reduction with surgical treatments based on pre-treatment gingival display at 6-month follow-up; Figure S6. Random-effects subgroup analysis of gingival display reduction with nonsurgical treatments based on pre-treatment gingival display at 6-month follow-up.
