# Supplementary material for: A Systematic Review and Meta-Analysis Comparing Surgical and Nonsurgical Treatments for Excessive Gingival Display
Source: Dent J (Basel). 2024 May 22;12(6):154. doi: 10.3390/dj12060154 (PMC11203257; doi:10.3390/dj12060154)
Supplement: Supplementary file 1 [file dentistry-12-00154-s001.zip › dentistry-2924717-SI.pdf]

dentistry journal

A Systematic Review and Meta-Analysis Comparing Surgical and Nonsurgical Treatments for Excessive Gingival Display

Mahdis Maleki [1], Bo Huang [1], Vanessa C. Mendes [1], Marco F. Caminiti [1] and Yoav Finer [1,2,*]

Table S1. Search keywords, MeSH terms, synonyms, and related concepts.

Population	Condition	Intervention	Primary Outcome	Secondary Outcome	Type of Study
Adult<br>Healthy<br>Human	EGD<br>EGD<br>Gingival smile<br>GS<br><br>Esthetics<br>Aesthetics	Gingival surgery<br>Gingivoplasty<br>Gingival contouring<br>Gingivectomy<br>Crown lengthening<br><br>Lip repositioning<br>Lip surgery<br><br>Orthognathic surgery<br>Le Fort 1<br><br>Botulinum toxin<br>Botox<br><br>Orthodontics<br>TADs<br>Skeletal anchorage<br>Intrusion	Outcome<br><br>Stability	Satisfaction<br>Quality of life<br><br>Complication<br>Complications<br>Morbidity<br>Adverse effects	Randomized controlled trial<br><br>Clinical trials<br><br>Non-randomized<br><br>Case control<br><br>Cohort<br><br>Prospective<br>Retrospective

Ovid MEDLINE: Epub Ahead of Print, In-Process & Other Non-Indexed Citations, Ovid MEDLINE® Daily and Ovid MEDLINE® <1946-Present>

#	Query	Results from 30 Jan 2023
1	((gummy or gingiv*) adj2 smile).tw,kf.	370
2	("excessive gingiv*" and (show or display or exposure)).tw,kf.	172
3	("smile esthetics" or "short upper lip" or "hypermobile lip" or "vertical maxillary excess").tw,kf.	422

4	smiling/	2,546
5	1 or 2 or 3 or 4	2,971
6	esthetics, dental/	13,494
7	oral surgery/	8,126
8	oral surgical procedures/	6,608
9	exp Minimally Invasive Surgical Procedures/	592,323
10	Gingiva/	19,199
11	Crown Lengthening/	549
12	exp Gingivoplasty/	1,781
13	exp Gingivectomy/	1,469
14	exp Lip/	12,558
15	myotomy/	623
16	Orthognathic Surgery/	1,117
17	exp Orthognathic Surgical Procedures/	6,786
18	Osteotomy, Le Fort/	2,422
19	botulinum toxins/ or botulinum toxins, type a/	18,302
20	orthodontics/ or orthodontic anchorage procedures/ or orthodontic appliances/ or orthodontics, corrective/	31,909
21	exp Tooth Movement Techniques/	9,682
22	("gingiva* surgery" or gingivoplasty or "gingiv* contour*" or gingivectomy or "crown lengthening").tw,kf.	1,443
23	("periodontal surgery" or "oral surgery" or "Orthognathic surgery" or "Nonsurgical therapy" or "maxillary intrusion").tw,kf.	13,866
24	(Lip adj2 (surgery or repositioning)).tw,kf.	473
25	("orthognathic surgery" or lefort or orthodont* or TAD* or "temporary anchorage" or botox or "botulinum toxin*").tw,kf.	83,418
26	BTX-A.tw,kf.	1,314
27	("case report*" or "case series").ti.	346,667
28	6 or 7 or 8 or 9 or 10 or 11 or 12 or 13 or 14 or 15 or 16 or 17 or 18 or 19 or 20 or 21 or 22 or 23 or 24 or 25 or 26	758,851
29	5 and 28	1,747
30	limit 29 to yr="2010 -Current"	1,138
31	30 not 27	1,060

32	limit 31 to ("reviews (maximizes sensitivity)" or "reviews (maximizes specificity)")	247
33	31 not 32	813

Embase Classic+Embase <1947 to 2023 January 27>

#	Query	Results from 30 Jan 2023
1	((gummy or gingiv*) adj2 smile).tw,kf.	358
2	("excessive gingiv*" and (show or display or exposure)).tw,kf.	161
3	("smile esthetics" or "short upper lip" or "hypermobile lip" or "vertical maxillary excess").tw,kf.	405
4	oral surgery/	22,812
5	Gingiva/	21,973
6	Crown Lengthening/	234
7	exp Gingivoplasty/	1,635
8	exp Gingivectomy/	1,617
9	lip/ or philtrum/ or upper lip/	22,147
10	myotomy/	4,029
11	Orthognathic Surgery/	5,047
12	Le Fort osteotomy/ or Le Fort I osteotomy/	1,029
13	botulinum toxin/ or botulinum toxin A/	43,243
14	orthodontics/ or orthodontic anchorage/ or orthodontic device/ or orthodontic procedure/	51,404
15	orthodontic tooth movement/	2,167
16	("gingiva* surgery" or gingivoplasty or "gingiv* contour*" or gingivectomy or "crown lengthening").tw,kf.	1,397
17	("periodontal surgery" or "oral surgery" or "Orthognathic surgery" or "Nonsurgical therapy" or "maxillary intrusion").tw,kf.	15,792
18	(Lip adj2 (surgery or repositioning)).tw,kf.	565
19	("orthognathic surgery" or lefort or orthodont* or TAD* or "temporary anchorage" or botox or "botulinum toxin*").tw,kf.	101,169

20	BTX-A.tw,kf.	1,913
21	("case report*" or "case series").ti.	452,819
22	4 or 5 or 6 or 7 or 8 or 9 or 10 or 11 or 12 or 13 or 14 or 15 or 16 or 17 or 18 or 19 or 20	212,540
23	1 or 2 or 3	755
24	22 and 23	625
25	24 not 21	557
26	limit 25 to yr="2010 -Current"	430
27	limit 26 to ("reviews (maximizes sensitivity)" or "reviews (maximizes specificity)")	140
28	26 not 27	290

Scopus:

TITLE-ABS-KEY ("gummy smile" OR "gingival smile" OR "excessive gingival show" OR "excessive gingival display" OR "excessive gingival exposure" OR "smile esthetics" OR "short upper lip" OR "hypermobile lip" OR "vertical maxillary excess") AND TITLE-ABS-KEY ("gingiva* surgery" OR gingivoplasty OR "gingiv* contour*" OR gingivectomy OR "crown lengthening" OR "periodontal surgery" OR "oral surgery" OR "Orthognathic surgery" OR "Nonsurgical therapy" OR "maxillary intrusion" OR "Lip surgery" OR "lip repositioning" OR "orthognathic surgery" OR lefort OR "le fort" OR orthodont* OR tad* OR "temporary anchorage" OR botox OR "botulinum toxin*" OR btx-a)) AND (LIMIT-TO (SRCTYPE , "j")) AND (LIMIT-TO (PUBYEAR , 2023) OR LIMIT-TO (PUBYEAR , 2022) OR LIMIT-TO (PUBYEAR , 2021) OR LIMIT-TO (PUBYEAR , 2020) OR LIMIT-TO (PUBYEAR , 2019) OR LIMIT-TO (PUBYEAR , 2018) OR LIMIT-TO (PUBYEAR , 2017) OR LIMIT-TO (PUBYEAR , 2016) OR LIMIT-TO (PUBYEAR , 2015) OR LIMIT-TO (PUBYEAR , 2014) OR LIMIT-TO (PUBYEAR , 2013) OR LIMIT-TO (PUBYEAR , 2012) OR LIMIT-TO (PUBYEAR , 2011) OR LIMIT-TO (PUBYEAR , 2010)) AND (LIMIT-TO (DOCTYPE , "ar"))

Cochrane Central

ID Search

#1 ((gummy or gingiv*) near/2 smile)

#2 "excessive gingival display"

#3 "excessive gingival exposure"

#4 "excessive gingival show"

#5 ("smile esthetics" or "short upper lip" or "hypermobile lip" or "vertical maxillary excess")

#6 MeSH descriptor: [Gingivoplasty] explode all trees

#7 MeSH descriptor: [Gingivectomy] explode all trees

#8 MeSH descriptor: [Lip] explode all trees

#9 MeSH descriptor: [Botulinum Toxins] this term only

#10 MeSH descriptor: [Oral Surgical Procedures] this term only

#11 MeSH descriptor: [Surgery, Oral] this term only

#12 MeSH descriptor: [Minimally Invasive Surgical Procedures] this term only

#13 MeSH descriptor: [Smiling] this term only

#14 MeSH descriptor: [Esthetics, Dental] this term only

#15 MeSH descriptor: [Gingiva] this term only

#16 MeSH descriptor: [Crown Lengthening] this term only

#17 MeSH descriptor: [Myotomy] this term only

#18 MeSH descriptor: [Orthognathic Surgery] this term only

#19 MeSH descriptor: [Orthognathic Surgical Procedures] explode all trees

#20 MeSH descriptor: [Osteotomy, Le Fort] this term only

#21 MeSH descriptor: [Botulinum Toxins, Type A] explode all trees

#22 MeSH descriptor: [Orthodontics] this term only

#23 MeSH descriptor: [Orthodontic Anchorage Procedures] this term only

#24 MeSH descriptor: [Orthodontic Appliances] this term only

#25 MeSH descriptor: [Orthodontics, Corrective] this term only

#26 MeSH descriptor: [Tooth Movement Techniques] explode all trees

#27 ("gingiva* surgery" or gingivoplasty or "gingiv* contour*" or gingivectomy or "crown lengthening")

#28 ("periodontal surgery" or "oral surgery" or "Orthognathic surgery" or "Nonsurgical therapy" or "maxillary intrusion")

#29 (Lip near/2 (surgery or repositioning))

#30 ("orthognathic surgery" or lefort or orthodont* or TAD* or "temporary anchorage" or botox or "botulinum toxin*")

#31 BTX-A

#32 #1 or #2 or #3 or #4 or #5 or #13

#33 #6 or #7 or #8 or #9 or #10 or #11 or #12 or #14 or #15 or #16 or #17 or #18 or #19 or #20 or #21 or #22 or #23 or #24 or #25 or #26 or #27 or #28 or #29 or #30 or #31

#34 #32 and #33 with Publication Year from 2010 to 2023, with Cochrane Library publication date Between Jan 2010 and Jan 2023, in Trials

Web of Science:

Results for "gummy smile" OR "gingival smile" OR "excessive gingival show" OR "excessive gingival display" OR "excessive gingival exposure" OR "smile esthetics" OR "short upper lip" OR "hypermobile lip" OR "vertical maxillary excess" (All Fields) AND "gingiva*

surgery" OR gingivoplasty OR "gingiv* contour*" OR gingivectomy OR "crown lengthening" OR "periodontal surgery" OR "oral surgery" OR "Orthognathic surgery" OR "Nonsurgical therapy" OR "maxillary intrusion" OR "Lip surgery" OR "lip repositioning" OR "orthognathic surgery" OR lefort OR "le fort" OR orthodont* OR tad* OR "temporary anchorage" OR botox OR "botulinum toxin*" OR btx-a (All Fields) and Article (Document Types) and 2010 or 2011 or 2012 or 2013 or 2014 or 2015 or 2016 or 2017 or 2018 or 2019 or 2020 or 2021 or 2022 or 2023 (Publication Years)

LILACS:

("gummy smile" OR "gingival smile" OR "excessive gingival show" OR "excessive gingival display" OR "excessive gingival exposure" OR "smile esthetics" OR "short upper lip" OR "hypermobile lip" OR "vertical maxillary excess") AND ("gingiva* surgery" OR gingivoplasty OR "gingiv* contour*" OR gingivectomy OR "crown lengthening" OR "periodontal surgery" OR "oral surgery" OR "Orthognathic surgery" OR "Nonsurgical therapy" OR "maxillary intrusion" OR "Lip surgery" OR "lip repositioning" OR "orthognathic surgery" OR lefort OR "le fort" OR orthodont* OR tad* OR "temporary anchorage" OR botox OR "botulinum toxin*" OR btx-a) AND (db:("LILACS") AND la:("en")) AND (year_cluster:[2010 TO 2023])

Table S2. Inclusion and exclusion criteria.

Category	Inclusion criteria	Exclusion criteria
Participants	>=18 years, male and female, any ethnicity, healthy, with chief complaint of EGD	< 18 years, smokers, or with any systemic condition that contraindicates treatment
Intervention	surgical or nonsurgical treatments for EGD	none
Comparison	pre-treatment condition, or a different method than the intervention	none
Outcome measures	primary outcome: change in the gingival display<br>secondary outcomes: patient satisfaction, complications	primary outcome not presented as mean change in gingival display, <6 months follow-up
Study design	randomized controlled trial, controlled clinical trial, non-randomized, non-controlled, prospective, retrospective, comparative cohort, and case-control	unpublished and non-peer-reviewed studies, review articles, books, expert opinions, animal studies, case reports, case series, clinical guidelines

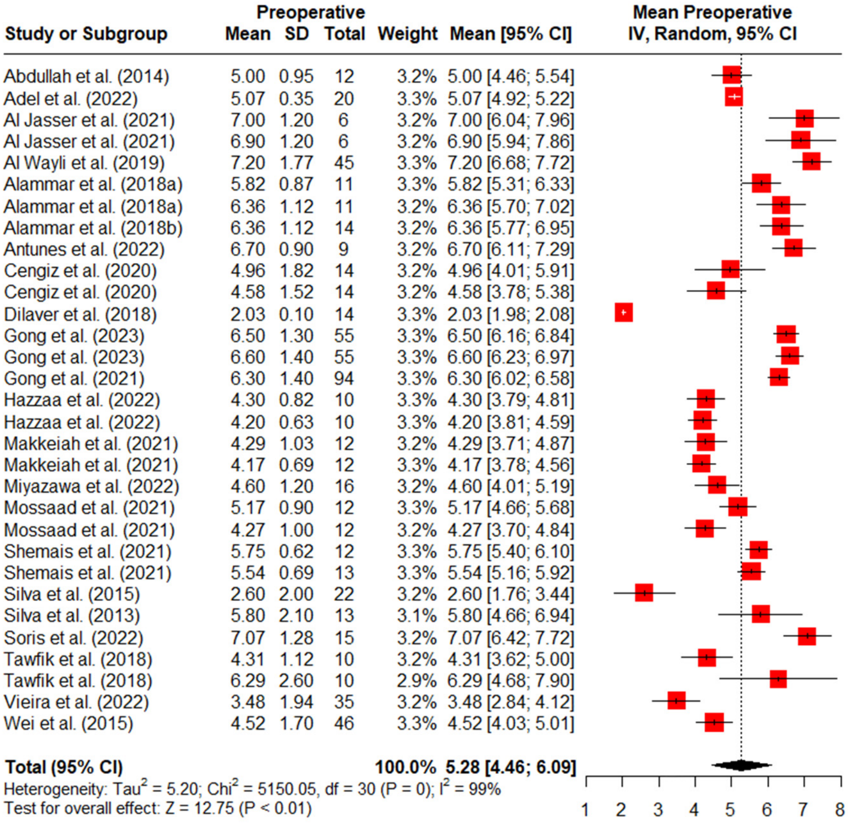

Figure S1. Random-effects meta-analysis of pre-treatment gingival display (SD: standard deviation; MD: mean difference; CI: confidence interval).

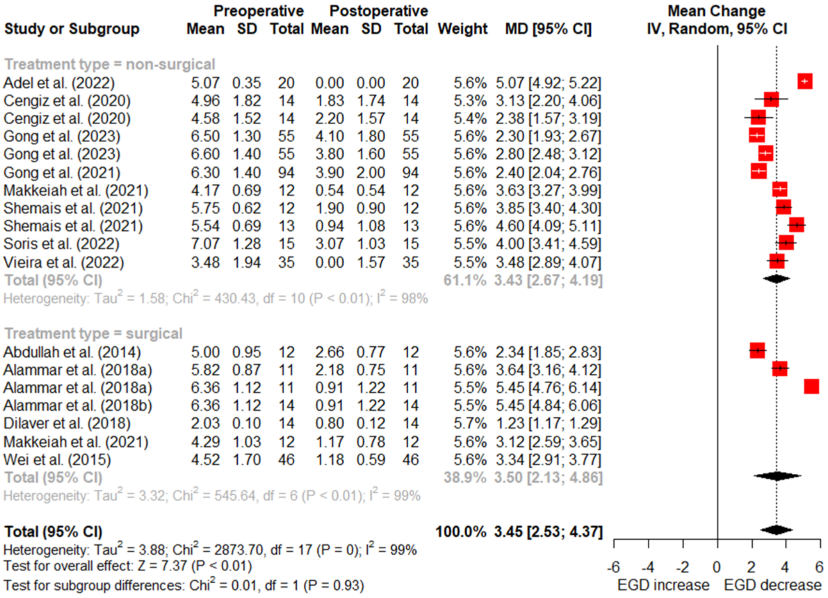

Figure S2. Random-effects meta-analysis of gingival display reduction with surgical vs nonsurgical treatments at 1-month follow-up (SD: standard deviation; MD: mean difference; CI: confidence interval).

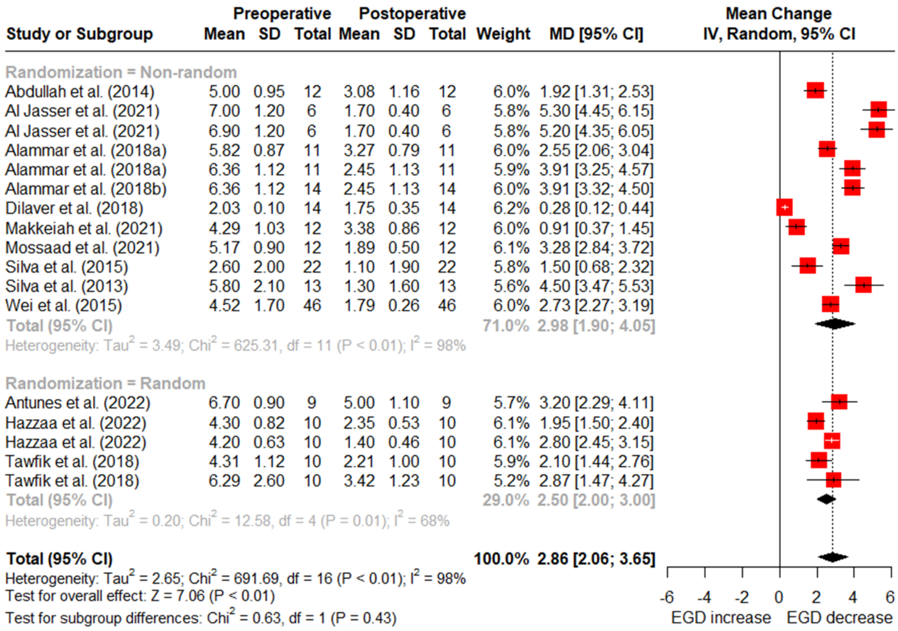

Figure S3. Random-effects meta-analysis of gingival display reduction with randomized vs non-randomized surgical treatments at 6-month follow-up (SD: standard deviation; MD: mean difference; CI: confidence interval).

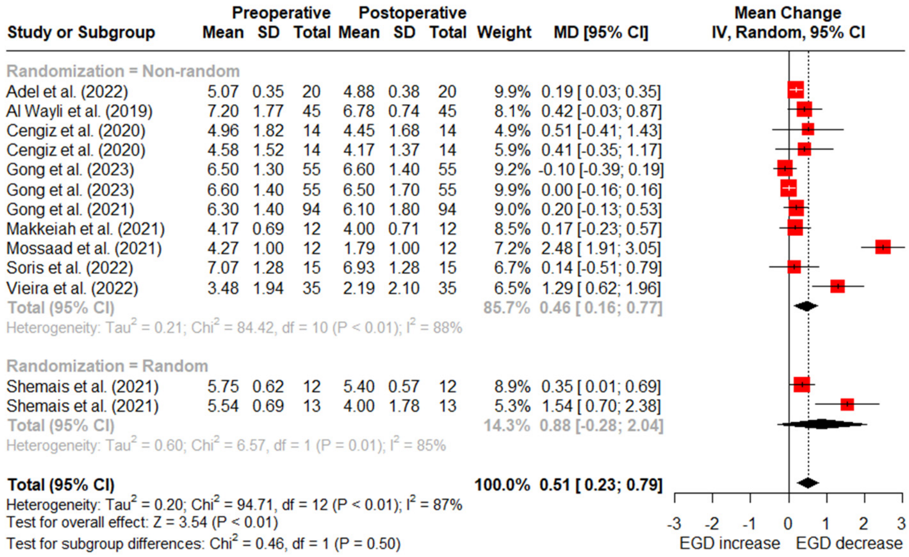

Figure S4. Random-effects meta-analysis of gingival display reduction with randomized vs non-randomized nonsurgical treatments at 6-month follow-up (SD: standard deviation; MD: mean difference; CI: confidence interval).

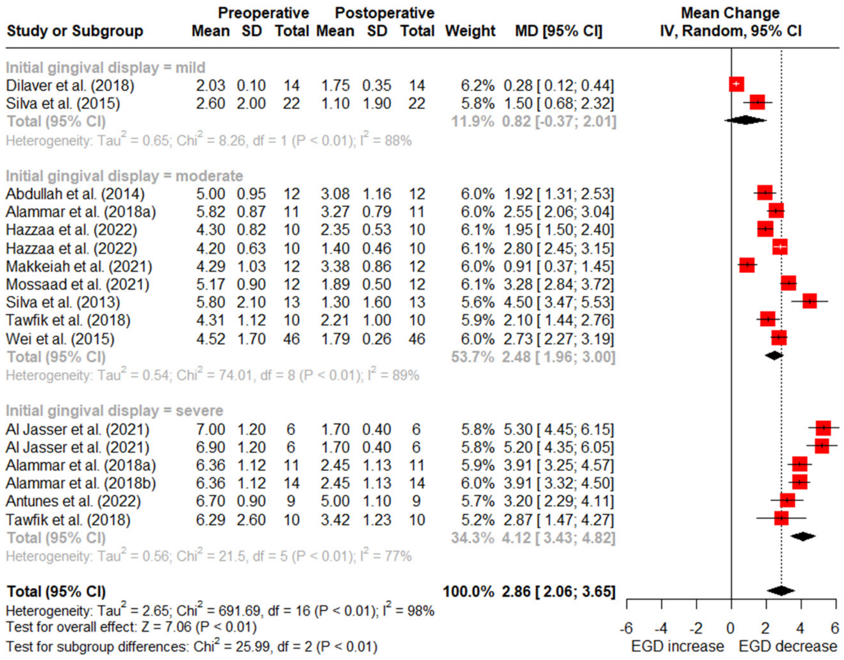

Figure S5. Random-effects subgroup analysis of gingival display reduction with surgical treatments based on pre-treatment gingival display, at 6-month follow-up (SD: standard deviation; MD: mean difference; CI: confidence interval).

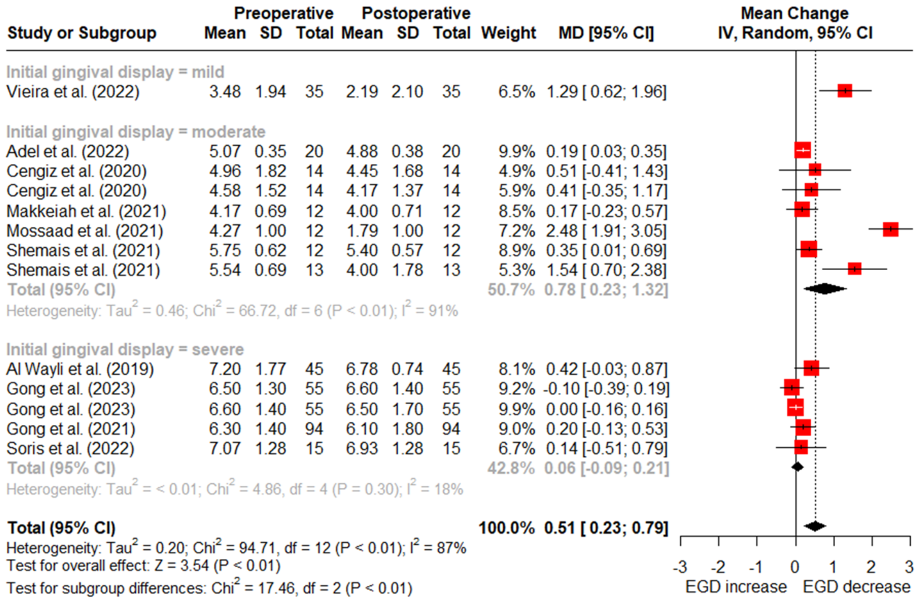

Figure S6. Random-effects subgroup analysis of gingival display reduction with nonsurgical treatments based on pre-treatment gingival display, at 6-month follow-up (SD: standard deviation; MD: mean difference; CI: confidence interval).
